# A static analysis approach for Android permission-based malware detection systems

**DOI:** 10.1371/journal.pone.0257968

**Published:** 2021-09-30

**Authors:** Juliza Mohamad Arif, Mohd Faizal Ab Razak, Suryanti Awang, Sharfah Ratibah Tuan Mat, Nor Syahidatul Nadiah Ismail, Ahmad Firdaus

**Affiliations:** Faculty of Computing, Universiti Malaysia Pahang, Pekan, Pahang, Malaysia; Sathyabama Institute of Science and Technology, INDIA

## Abstract

The evolution of malware is causing mobile devices to crash with increasing frequency. Therefore, adequate security evaluations that detect Android malware are crucial. Two techniques can be used in this regard: Static analysis, which meticulously examines the full codes of applications, and dynamic analysis, which monitors malware behaviour. While both perform security evaluations successfully, there is still room for improvement. The goal of this research is to examine the effectiveness of static analysis to detect Android malware by using permission-based features. This study proposes machine learning with different sets of classifiers was used to evaluate Android malware detection. The feature selection method in this study was applied to determine which features were most capable of distinguishing malware. A total of 5,000 Drebin malware samples and 5,000 Androzoo benign samples were utilised. The performances of the different sets of classifiers were then compared. The results indicated that with a TPR value of 91.6%, the Random Forest algorithm achieved the highest level of accuracy in malware detection.

## Introduction

The use of mobile devices has rapidly increased throughout the world in recent decades, with most people now owning mobile device. The convenience of mobile devices enables many online activities to be performed, for instance, the online streaming of information, social networking, video viewing, and online banking. This proliferation of technology has also provided opportunities for the deployment of malware codes designed to target mobile devices. Malware is malicious software that attacks the files or programmes that are stored within mobile devices. Malware can be classified according to the mechanism by which it gains access to a system: worms, backdoors, trojans, rootkits, spyware, and adware [1]. The McAfee Report [2] noted that malware such as backdoors, crypto mining, fake applications, and banking trojans increased substantially in the latter half of 2019. Hidden applications and adware were noted to be the most common form of mobile threats in the Android operating system. The McAfee report also indicated that the incidence of malware attacks is increasing every year, with over 30 million mobile malware attacks detected in 2018.

**Fig 1:**
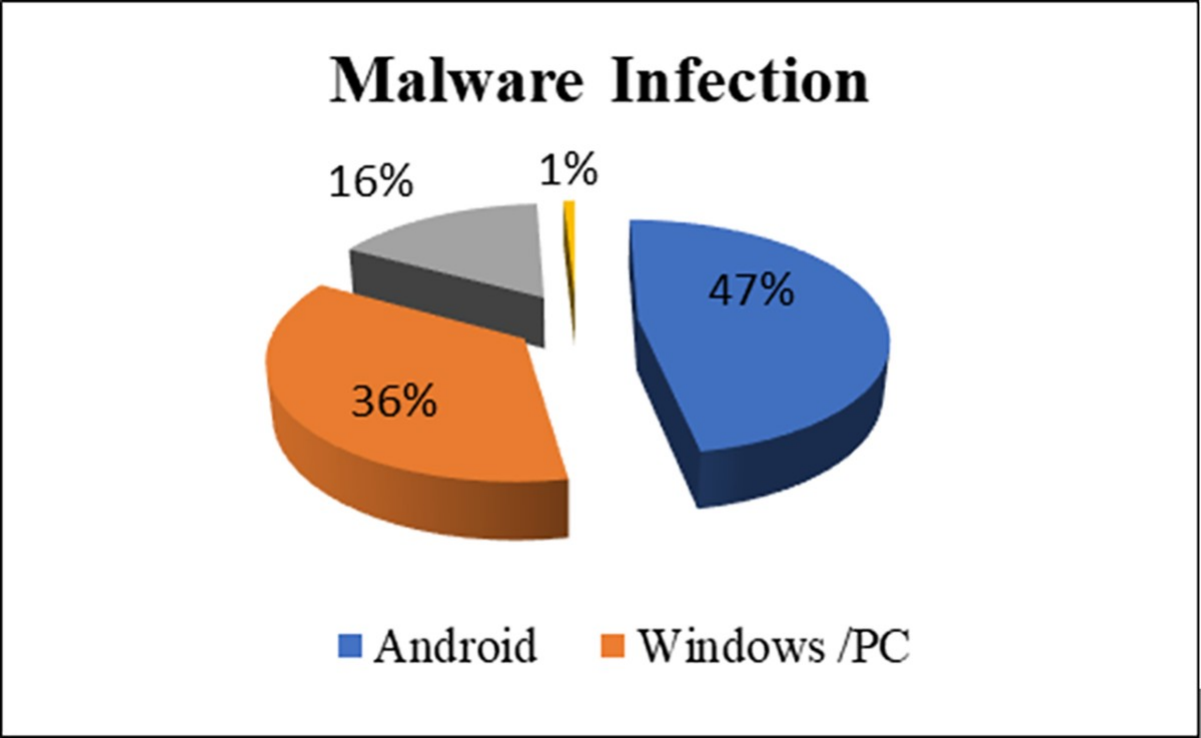
Malware attacks in 2018 by mobile device.

Among the various mobile devices available, Android mobiles are the most commonly targeted by malware. The International Data Corporation (IDC) report on worldwide shipments stated that Android mobiles are leading the market, with an increase from 85.1% in 2018 to 87.0% in 2019. In 2020, despite showing a slight decline, Android still led the market at 84.1% compared to iOS 15.9% and others 0% [3]. However, the popularity of Android mobiles has produced more security concerns because it raises the prospect of more threats from attackers who spread the malware, which causes the application to act maliciously. Android devices have been identified as the most highly targeted systems, with the highest percentage of malware infections at 47.15%. Malware typically works initially by sending fraudulent messages to users. When users become interested in these messages, they are charged for fake services. Other systems tend to suffer less threats: In 2018, the threat rate for Windows/PCs was 35.82%, the Internet of Things (IoT) was 16.17%, and iPhones had a threat rate of 0.85% [4]. Fig 1 shows the percentage of malware attacks on various mobile device in 2018.

**Fig 2:**
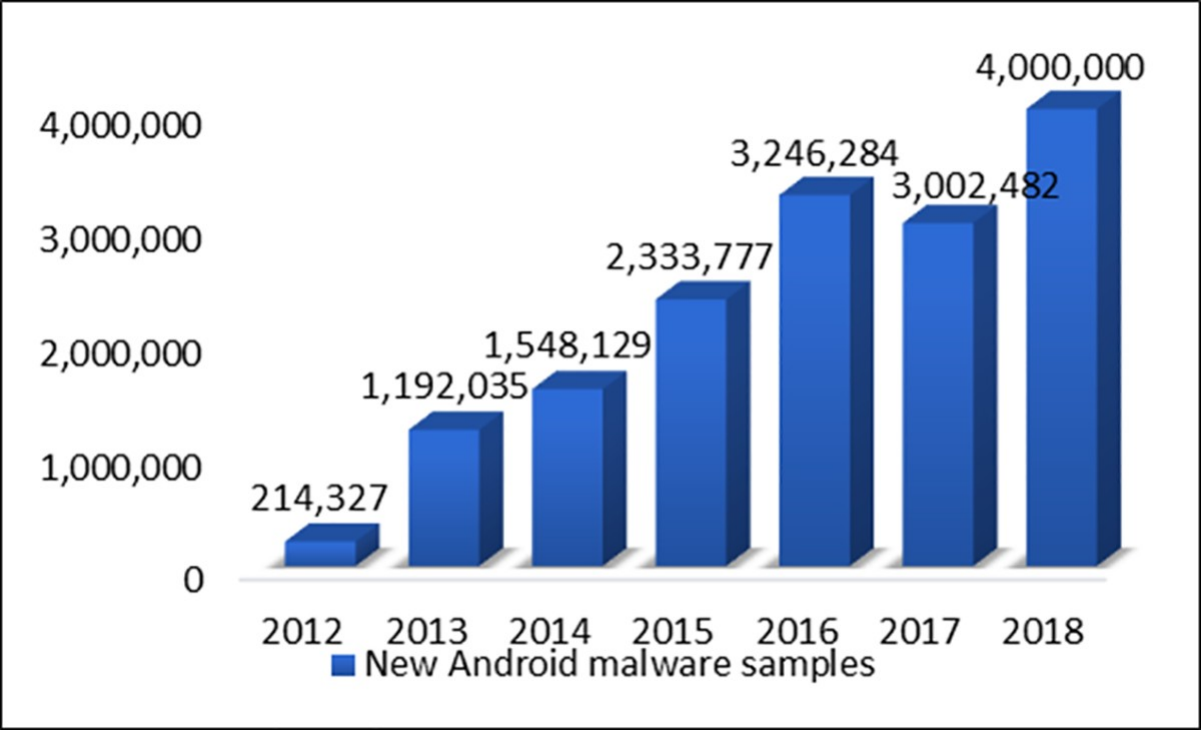
New Android malware samples 2012–2018.

A G DATA Cyber Defence analysis showed that a total of 3.2 million new malware were detected in the third quarter of 2018 in contrast to the third quarter of 2017, in which 2,258,357 malware were detected [2]. The use of Android malware has risen to an extraordinary level. Fig 2 shows the drastic increase in new Android malware samples from 2012 to 2018.

In the first half of 2019, it was observed that the number of Android malware attacks was 1.85 million and increased to 4.18 million by the end of 2019 [3]. These malware attacks tend to be surreptitious and creep insidiously into the mobile system. To avoid malware attacks, mobile users need to take several steps: Avoid installing applications from unknown sources, avoid downloading applications from unregistered websites, and foster the habit of using antivirus software [4]. Antivirus software offers a system for detecting and preventing malware. However, the weakness of the existing commercial tools is that they only provide protection against known malware, and unknown or new malware remains undetected [5]. Any mobile device that is monitored by antivirus software needs frequent signature updates to detect new malware. Usually, antivirus software prompts the user to install an update to maintain the security of the device. However, these processes degrade the memory and power consumption performance of devices [6].

Existing Android security, such as Bouncer, scans and blocks permission requests from various applications automatically [7]. It is available for download via the Google Play Store. However, users are at risk of malware attacks if the application is downloaded from a third-party source. The Android operating system does not govern third-party sources, and users should exercise caution when agreeing to unreasonable permission requests during the application installation process [8]. Android applications that request more permissions than needed can transform the application’s origin from benign to malicious [9].

Additionally, for Android security, another approach is a permission-based system that acts as a firewall, filtering out all applications that require permission and preventing Android applications from accessing personal data [10]. When the application is installed on Android, the user is notified of the permission request, which should be handled carefully. Even so, when users grant permissions without fully comprehending the risks, they expose themselves to unsafe conditions and malicious attacks. An unscrupulous programmer may take advantage of the user to perform various malicious actions without the user’s consent. Another security mechanism that protects users from malware attacks is an intrusion detection system (IDS). Usually, an IDS monitors the network or system to detect intrusions and analyse data traffic [11]. IDS is widely used in different fields as one of the security mechanisms. It is also used in the CAN Bus system, where researchers propose CANintelligentIDS to enhance security features to prevent malware threats [12, 13] review the IDS on clone node attacks in a static wireless sensor network. The IDS protects mobile devices against known or unknown malware; it also ensures that user data are confidential [10]. Nonetheless, the proliferation of Android malware demonstrates that there is still room for research into the most effective methods for countering malware attacks.

Previous research has presented various approaches in mobile malware detection for Android mobile users [7, 14, 15]. Static and dynamic analyses are two common techniques used in malware detection [16, 17]. Both techniques are popular as they are perceived to be successful analysis methods capable of defending Android applications. The static analysis focuses on detecting Android malware by examining a file without running it on the system. This technique includes signature and component-based analyses, reverse engineering, and Dalvik bytecode. Meanwhile, dynamic analysis is capable of detecting malware based on the behaviour observed in an isolated environment, such as a simulator and virtual machine [15]. Both techniques are then combined to form a hybrid model for improving malware detection and identification. The hybrid analysis technique performs the static and dynamic analyses separately; hence, it consumes more resources and takes longer to complete the analysis [18].

An alternative technique is machine learning analysis. This technique automates the analysis of mobile malware detection by recognising the malware pattern. It can achieve a high rate of malware detection. This technique is a specific field of artificial intelligence that predicts future decisions and outputs based on datasets. It refers to the process of characterising malware behaviour and applying classifiers to evaluate the dataset [19]. In previous studies, the classifiers most frequently used to assess the necessary features and malware detection include Naive Bayes, support vector machines, decision trees, Random Forest, K-means, K-nearest Neighbours AdaBoosting, logistic regression, and J48 [20]. The machine learning and static analysis techniques require features used to compare the malware and benign applications to obtain accurate detection. Features used in related studies for malware detection include the Application Programme Interface (API), a function call, code structure, AndroidManifest.xml, Intent, and permission [9]. The malware datasets were usually retrieved from Contagiominidump, Genome, Drebin, AMD, and VirusShare, while the benign datasets were usually retrieved from Google Play, APKpure, APKsapk, Playdrone, and Androzoo.

This study proposes a malware detection system to scrutinise new known malware variants; it also aims to identify insecure permissions existing in mobile applications. In that respect, the current study uses static analysis as an approach by focusing on the manifest file (AndroidManifest.xml), which consists of specific permissions geared towards Android application development. Such permissions are incapable of change once the development is completed. In the current study, the features in the manifest file are first organised into a table before the classification process. An optimisation approach is then utilised to demonstrate the most suitable features to detect the malware. This study attempts to evaluate the efficacy of the static analysis technique by using the machine learning approach and using the application’s permission noted in the manifest file.

The following are the paper’s primary contributions:

- To identify the optimal features via optimisation techniques in order to improve Android malware detection accuracy.
- To conduct an experiment using Android permission features by implementing static analysis in the Android malware detection.
- To assess the machine learning classifier’s effectiveness at detecting Android malware.

The rest of the paper is organised as follows: Section 2 presents the state of the art of malware detection. It introduces the previous research conducted in the field of Android mobile malware detection. Section 3 discusses the methodology of the study. It describes the general process of implementing an Android malware detection system, such as the general architecture, the data collection phase, and the machine learning phase. Section 4 presents the evaluation and results of the implementation. It focuses on the evaluation of the experiment and the efficacy of the proposed method. Section 5 provides the conclusion. It explains the achievement of the objectives and the contributions of this research to malware detection. It also discusses the findings and limitations of this study.

## Related work

In this era of globalisation, people commonly use their mobile devices in a variety of ways, for example, as a network connection to interact with the world, for online shopping, for online banking transactions, or even for cloud storage of files and documents. Nonetheless, some unscrupulous programmers take advantage of these situations to create and install mobile malware. Mobile malware is capable of stealing user information and exploiting it [21]. There are many ways in which malware can corrupt the system or programmes of mobile devices, such as bypassing the access control of the devices and deleting or encrypting sensitive data [15], consuming excessive battery [20], and even turning the device into a botnet zombie [1]. Malware is defined by its malicious contents and behaviour that violates the requirements of the system. This includes observing user system activities without authorisation. Malware spreads by self-propagating and through social engineering [20]. Self-propagating strategies automatically install malware into a mobile device, for instance, worms and viruses [22]. Social engineering takes advantage of users who have no security knowledge. It tricks such users into manually installing malware applications onto their mobile devices [20]. Cabir was the first malware detected in 2004 that could use networking technologies to spread and generate infections [10]. The Zeus botnet, which was reported in 2007, is one of the common malware that can control many computers and steal data in banking organisation [23]. The DroidDream is the mobile malware detected in 2011, and it can gain root access, steal information and add malware to the mobile phone [24, 25]. FakePlayer is Russia’s first Trojan Android malware discovered in 2010. It works as a media player application and sends the SMS to the premium number without user consent [10].

The Android system is an open system, which can become an easy target for malware attackers who can contaminate the operating system [26]. In May 2019, Android malware tracking recorded more than 10.5 million programmes [27]. The growth of Android mobile users has certainly augmented the spread of Android malware. As can be observed in the Nokia Threat Intelligence Report [28], of the 20 most frequently detected Android malware in 2018, six were new malware. Android.Adware.AdultSwine was the most frequently detected Android malware at 17.29%. AdultSwine uses inappropriate advertisements to deceive mobile users into installing and registering fake security applications with an unknown cost. Once this installation has been performed, the malware is very difficult to remove [4]. In 2017, Android.Adware.Uapush.A was listed as the leading Android malware. Android.Adware.Uapush is an Android adware Trojan that has had a moderate impact on mobile users. The Uapush adware sends the International Mobile Subscriber Identity, the International Mobile Equipment Identity, contact information, bookmarks, and call history to a Command and Control Server in China [29]. The adware then sends a message with obscure programming to advertise and promote a business without user consent [30]. Even though the Uapush adware was the highest ranking in terms of attacks in 2017, its impact on mobile users has not been too severe.

### Malware intrusion detection systems

**Fig 3:**
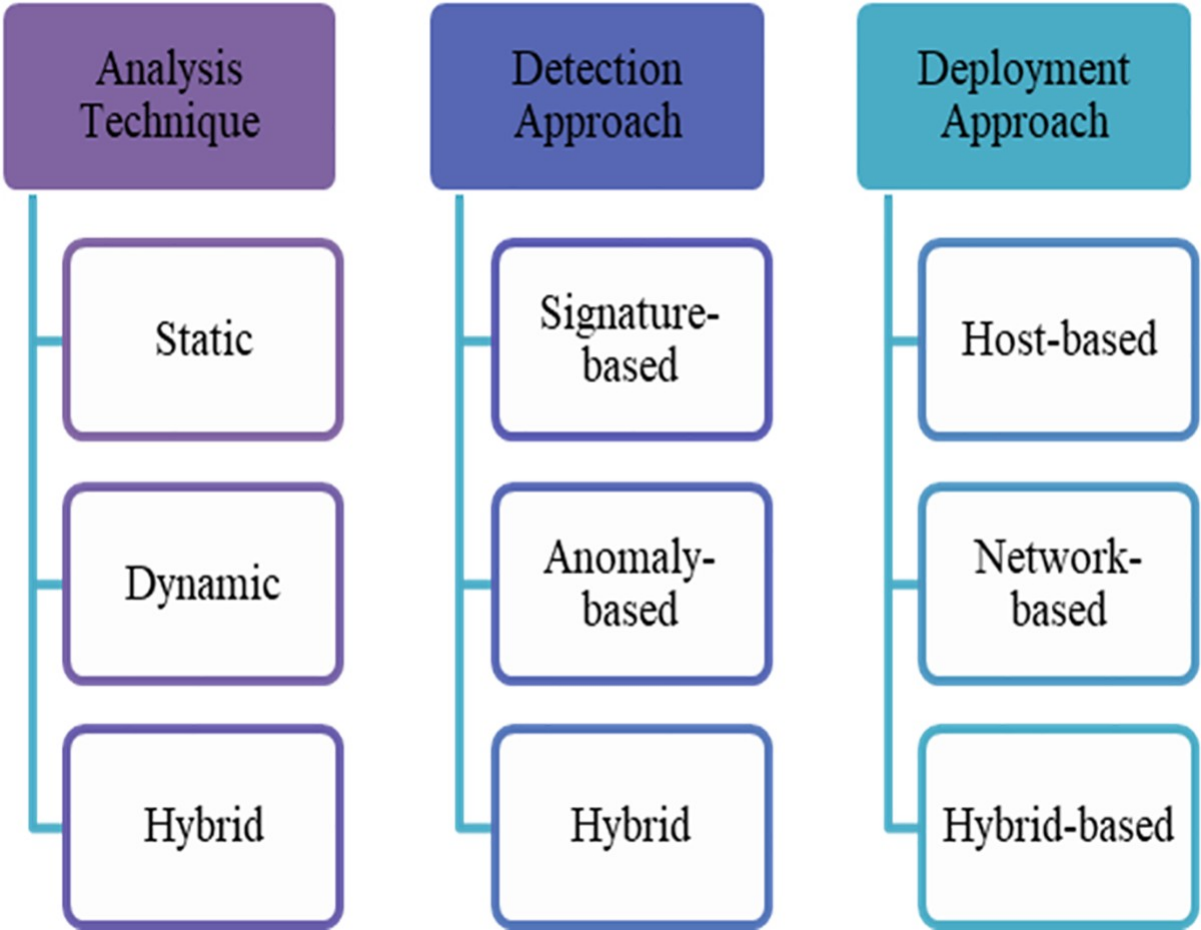
Taxonomy of malware intrusion detection systems.

Although there are security mechanisms, such as firewalls, antivirus software, and IDSs, to secure mobile devices, there is still a need to develop a novel approach towards detecting malware. Commercial antivirus mechanisms can effectively detect known malware, but they are incapable of detecting unknown malware. Therefore, malware detection techniques with high levels of accuracy and speed are important to ensure the effectiveness of such malware applications. The IDSs were developed to protect devices from attackers. If an intrusion is detected, then it is logged, and an alert is generated. The malware IDSs are divided into three classifications, as illustrated in Fig 3.

#### Analysis technique

The two most common techniques used in this field are static and dynamic analysis techniques. The static analysis technique analyses the programmes without executing them. The static examination is possible by using the program analyser, debugger, and disassembler [31]. The features commonly used in the static analysis are permission, intent filters, Java code, network address, strings, and hardware components [32]. Android applications use permissions to protect the mobiles from malware. The application requests permission to access the mobile device during the installation process [33]. Meanwhile, Android applications use API calls to communicate with the devices. Dishonest programmers normally change the sequence and then rename the API calls to evade the detection system. This is known as code obfuscation. It is then saved as a Dalvik format. Fraudulent programmers are able to modify the code-base and inject malicious code into Android applications. All the static features are accessible in the AndroidManifest.xml file or Java code file. Table 1 shows the related work in static analysis.

**Table 1:** Related work in static analysis.

References	Year	Tools	Objective	Limitation
[32]	2009	Kirin	To provide lightweight certification for applications that incorporate security rules to mitigate malware during the installation process.	Specific rules may be deemed unenforceable for any number of reasons.
[33]	2011	Stowaway	To detects overprivileged in compiled Android applications.	The tool is unable to handle multiple reflective calls.
[34]	2012	RiskRanker	To analyse a specific application dangerous behaviour using risk analysis	Malware is easy to escape in the first step, as RiskRanker uses the heuristic only in the second step
[35]	2013	AndroSimilar	To generates a signature by extracting statistically improbable features to detect malicious Android apps.	Consist significantly less malware signature
[36]	2014	FLOWDROID	To provides static taint analysis for Android applications	Resolves reflective calls only with string constants and unaware of multi-threading
[37]	2020	AdDroid	To analyse and detect malware in Android applications using a variety of artefact combinations referred to as Rules.	The small and imbalanced dataset is used in the experiment

In contrast, the dynamic analysis runs the application in a safe environment while observing the malware behaviour [7]. It is capable of detecting malware when the obfuscation technique is applied. The features most commonly selected for detecting malware in the dynamic analysis were memory and registry usage, instruction traces, network traffic, and API call traces [38]. More than 1,000 datasets were used in these studies. The accuracy rate of the detection was more than 90%. Various tools used in the dynamic analysis include CrowDroid, TaintDroid, ParanoidAndroid, Aurasium, Appfence, and DriodScope [17]. The hybrid analysis combines both static and dynamic analyses. In the hybrid analysis, user behaviours and permission intent were the most common features selected for detecting malware. More than 4,000 datasets extracted from MARVIN, Drebin, Genome and Virushare were used in these studies. The accuracy rate of the detection was more than 90% [39].

#### Detection approach

There are two common approaches used for detecting malware: Signature-based and anomaly-based [40]. The signature-based approach relies on recognising the signature of the malware behaviour. In contrast, the anomaly-based approach uses its knowledge to compare the normal and abnormal behaviours of a system [1]. Programmes that deviate from the specifications are assessed as anomalous and, usually, as malware. The combination of these two approaches is known as a hybrid approach.

#### Deployment approach

The deployment approach in the IDS can be divided into three categories: A host-based intrusion detection system (HIDS), a network-based intrusion detection system (NIDS), and a hybrid-based IDS. The HIDS assembles resources from end devices (host) and servers. It also monitors and analyses intrusive traffic in the system resources and emphasises the CPU consumption, the file system, memory, and device [30]. In the event of a change occurring to the host system, the IDS sensor then checks the abnormal activity in the log entry by using a signature. If the signatures correspond, the sensor will notify the management console [41]. If any anomalous activities have occurred, the administrator will receive a warning. Another development of the HIDS is the application-based IDS. It monitors networks’ traffic, such as inspection files, void file executions, and abnormal traffic.

A NIDS is used to sniff network traffic for examination. This is accomplished by using a deep packet analyser [1]. A NIDS is vital in today’s computer network infrastructure to monitor and identify unwanted and malicious network traffic [22]. The NIDS uses anomaly- and signature-based detection approaches. The sensor in the NIDS analyses all the packet headers to detect malicious attacks; HIDS cannot detect this type of attack [42]. Analysis of the header packet can detect the IP-based denial of service attacks in network traffic. The NIDS sensor is capable of detecting attacks based on the packet header in real-time. Many tools are used in the NIDS. Among them are Honeypot, Snort, Solarwinds, Sagan, and Splunk. The combination of the HIDS and NIDS approaches is known as a hybrid-based IDS.

#### Android malware features

**Fig 4:**
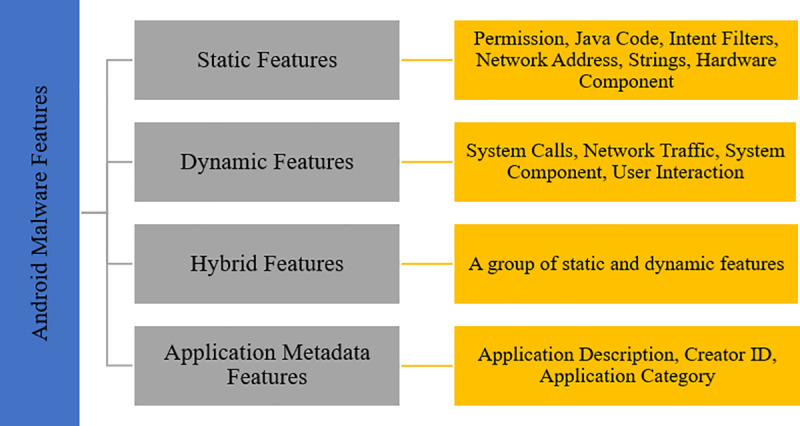
Taxonomy of Android malware features.

Android malware features include static, dynamic, hybrid, and application metadata features. Fig 4 shows the taxonomy of Android malware features.

The static features are available in the AndroidManifest.xml or Java code file. The common static features used are permission, Java code, intent filters, network address, strings, and hardware components. In comparison, dynamic features refer to the application behaviours that communicate between the operating systems or networks. A previous study used network traffic, system calls, and API calls as dynamic features to detect malware. Hybrid features are those derived from a combination of static and dynamic features. They are used in the detection system as an additional measure to increase accuracy. The application metadata features refer to the information that appears before a download installs the application. They include the application description, rating, requested permission, and developer information. The selection of features is a crucial part of the classification process. Some of the features used in previous studies [43–45] were permission-based features [40] that used the API call sequence features and [46] intent.

The current study applied permission-based features (static features) to detect Android mobile malware. Permission codes involve applications making frequent permission requests, such as for the internet, to send_sms, access_network_state, receive_sms, and write_external_storage. In this regard, mobile users should be aware of the application permission request to be better prepared for protecting their mobile devices. Neglecting an application’s permission request can cause harm to mobile devices. Android permissions are the permissions that an application requests the mobile user to grant during the installation. It is the first security step in Android mobile devices. The permission is the first obstacle a user encounters with an unscrupulous programmer before an attack. Android permissions can be categorised into four levels of protection known as a normal, dangerous, signature, and signatureORsystem [19, 47]. Each protection level involves a base permission type and zero or more flags. Normal permissions are the default permissions of lower risk, which are automatically granted during installation without the user’s permission. Dangerous permissions are higher risk permissions that allow a malware application’s request to access the user’s data or control devices, exposing mobile users to threats. Signature permissions automatically grant the permission of the application request when a signed certificate matches the application that declared the permission. SignatureOrSystem grants the applications in a dedicated folder on the Android system image or that are signed with the same certificate as the application that declared the permission. Multiple vendors use SignatureOrSystem level to share specific features when developing applications. Therefore, the awareness of mobile users concerning the dangers of malware must be increased. Otherwise, more damage and losses to their devices will occur [1]. Table 2 shows the protection level of Android permissions, descriptions, and examples of the permissions [47].

**Table 2:** Android permission protection level.

Protection level	Description	Example of permission features
Normal	Low risk to users or apps. Automatically allow the permission, and the user did not revoke the permission	ACCESS_LOCATION_EXTRA_COMMANDS, ACCESS_NETWORK_STATE, ACCESS_NOTIFICATION_POLICY, ACCESS_WIFI_STATE.
Dangerous	High risk to the user. Apps need to prompt the user and wait until user approval	ACCESS_MEDIA_LOCATION, ACCESS_FINE_LOCATION, ACCESS_BACKGROUND_LOCATION, ACCEPT_HANDOVER.
Signature	The system grants the apps when the same certificate signs the apps	BIND_ACCESSIBILITY_SERVICE, BIND_AUTOFILL_SERVICE.
SignatureOrSystem	Grants the applications in a dedicated folder that signed with the same certificate.	BATTERY_STATS BIND_CALL_REDIRECTION_SERVICE

## Methodology

**Fig 5:**
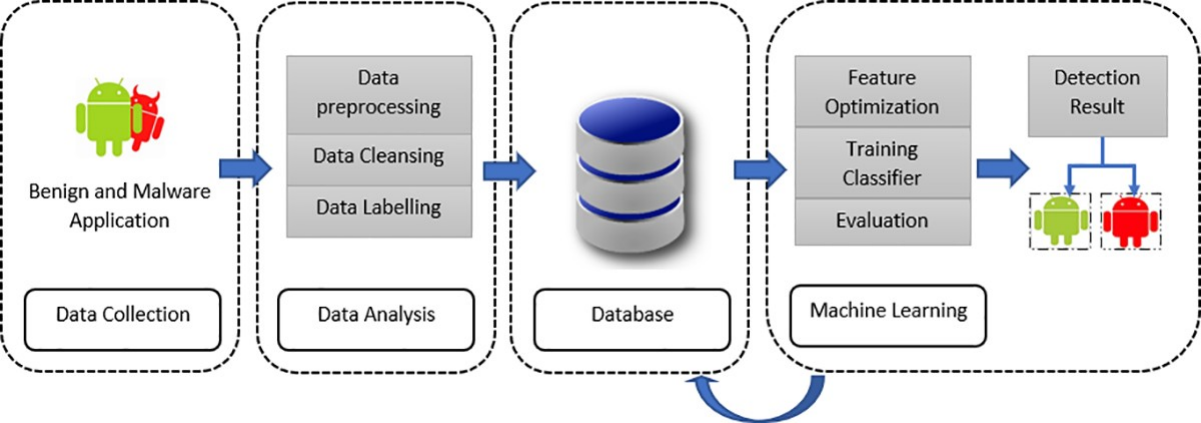
Malware detection system.

This section discusses the process of the malware detection system and the implementation of machine learning. There are four phases in the Android mobile malware detection system. They include data collection, data analysis, the database, and machine learning. The fourth phase contains the evaluation phase, which uses the machine learning approach to classify each of the malware families and analyse the application permissions that can detect benign or malware applications. Fig 5 shows the architecture of the malware detection system.

Detailed in the figure is the malware detection system process. It involves the four phases mentioned above. The data collection begins by collecting all the permissions in the malware and benign applications. The data are then processed in the analysis, which decompiles the apk file. This is then extracted to the data cleansing process, which filters the permissions. For data labelling, all the permissions that have been assembled are saved in an attribute relation file format (x.arff). They are then stored in a database in a readable format. All the feature attributes used in the feature optimisation process are saved in a.arff file. A three feature optimisation technique aims mainly at analysing patterns.

The data collection and the optimisation process of the features are crucial for detecting malware. During the cleansing process, malware and benign behaviours are detected. Following this, a notification is then sent to the database. During this phase, data labelling relies on the permission package name to ensure that the same features and applications are detached from the database. The final process is machine learning, whereby feature optimisation executes the labelled data with 10-fold cross-validation and evaluation. This is achieved by using a training classifier to detect the malware. The current experiment utilises the detection performance, such as the level of accuracy and false positive rate, to detect malware.

### Data collection process

In the data collection phase, the malware and benign datasets were extracted and then compiled in.csv file format. The random samples were retrieved from the Androzoo and Drebin datasets. The Androzoo dataset consists of more than three million applications and is scrutinised by ten types of antivirus products for malware detection. This dataset has the potential to contribute to new research topics within the Android application and can engage in reproducible experiments. The dataset is accessible at https://Androzoo.uni.lu [48]. The Drebin dataset contributes to the public academic dataset of Android malware that was launched in 2014. The dataset was introduced in the article ‘DREBIN: Effective and Explainable Detection of Android Malware in Your Pocket’. The research community mostly uses the Drebin dataset as a malware dataset to evaluate the effectiveness of a detection system and compare an algorithm’s performance. The Drebin dataset is available at the website Http://user.cs.uni-goettingen.de/˜darp/drebin [49]. A set of 10,000 samples from the dataset that is confined to the applications is presented in Table 3.

**Table 3:** Dataset summary.

Dataset	Source	Total Dataset
Benign	Androzoo	5000
Malware	Drebin	5000
**TOTAL**		10,000

**Fig 6:**
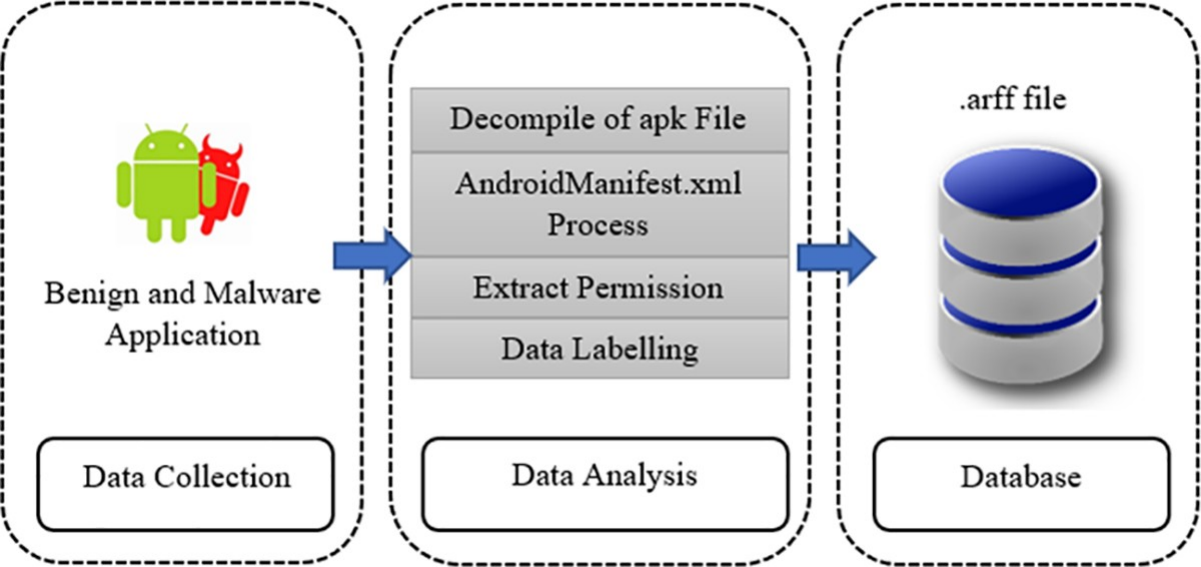
Data collection process.

The benign applications were downloaded from Androzoo, which belongs to the Google Play store. The malware applications were downloaded from the Drebin project. The current study collected 274 lists of permissions (known as features). The datasets were then labelled as benign or malware in the last column. The 10,000 samples were indicated by hash numbers to avoid duplications. The datasets were then saved in a binary format (.CSV) and subsequently transformed to the attribute relation file format. Fig 6 shows the data collection process.

The AndroidManifest.xml file was used to obtain essential information such as the permissions and activities of the application. Before the dataset were saved in the database as a x.arff file, all extracted permissions were labelled as benign and malware. These features served as attribute values and were termed the ‘feature set’. The values represent the binary value concerning whether permission was requested or not by the application. The values were stated as ‘1’, which represented permission requested by a specific application, while ‘0’ represented permission not requested by the specific application. Table 4 shows the 10 Android applications most frequently granted permissions for access to the system by malware or benign applications analysed from the database.

**Table 4:** 10 most frequent permission requests in malware and benign applications.

Malware Application			Benign Application		
Permission	Protection Level	Frequency (%)	Permission	Protection Level	Frequency (%)
INTERNET	Normal	99	INTERNET	Normal	81
READ_PHONE_STATE	Dangerous	94	ACCESS_NETWORK_STATE	Normal	74
WRITE_EXTERNAL_STORAGE	Dangerous	72	WRITE_EXTERNAL_STORAGE	Dangerous	55
ACCESS_NETWORK_STATE	Normal	71	WAKE_LOCK	Normal	32
SEND_SMS	Dangerous	53	READ_PHONE_STATE	Dangerous	31
RECEIVE_BOOT_COMPLETED	Normal	52	VIBRATE	Normal	25
ACCESS_WIFI_STATE	Normal	48	ACCESS_WIFI_STATE	Normal	24
WAKE_LOCK	Normal	42	ACCESS_FINE_LOCATION	Dangerous	22
RECEIVE_SMS	Dangerous	40	GET_ACCOUNTS	Dangerous	22
READ_SMS	Dangerous	40	RECEIVE	Signature	21

**Fig 7:**
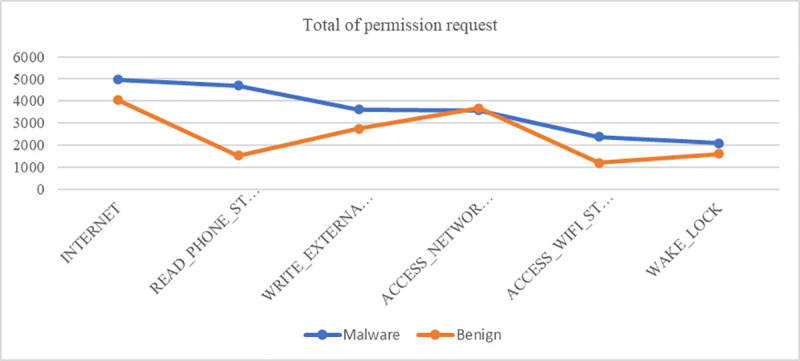
Comparison of total permission requests by malware and benign applications.

Table 4 shows that the malware and benign applications had six comparable permissions involving INTERNET, READ_PHONE_STATE, WRITE_EXTERNAL_STORAGE, ACCESS_NETWORK_STATE, ACCESS_WIFI_STATE, and WAKE_LOCK. The INTERNET permissions comprised the highest percentage, with malware at 99% and benign at 81%. Seven applications were dangerous permissions involving READ_PHONE_STATE, WRITE_EXTERNAL_STORAGE, SEND_SMS, RECEIVE_SMS, READ_SMS, ACCESS_FINE_LOCATION and GET_ACCOUNTS. Table 5 shows the description of the ten permission features most requested by malware applications. Fig 7 shows the comparative graph for the six permissions requested by the malware and benign applications.

**Table 5:** Description of the ten highest permission requests by malware applications.

Android Application	Description
INTERNET	Enable apps to open the network socket.
READ_PHONE_STATE	Enable access to phone state with read-only privilege (phone number, ongoing calls status, cellular network information, and PhoneAccounts).
WRITE_EXTERNAL_STORAGE	Enable external storage written by any apps
ACCESS_NETWORK_STATE	Enables apps to access network information
SEND_SMS	Enables apps to send Short Message Service (SMS).
RECEIVE_BOOT_COMPLETED	Enables apps to accept the broadcast Intent.ACTION_BOOT_COMPLETED after the complete boot process.
ACCESS_WIFI_STATE	Enables apps to grant information in Wi-Fi networks.
WAKE_LOCK	Enables use of PowerManager WakeLocks to manage sleeping processor or dimming screen.
RECEIVE_SMS	Enables apps to receive SMS messages.
READ_SMS	Enables apps to read SMS messages.

The graph indicates that INTERNET applications made the highest number of requests. Although the INTERNET application request was the highest, it did not have a critical impact on mobile users because the protection level is normal. The READ_PHONE_STATE permission had the highest percentage of requests among the dangerous applications requested by malware applications. This permission allows an unscrupulous programmer with privileged read-only access to phone state information, such as network information, phone numbers, call history, and the phone account [49]. As a result, the mobile user needs to pay more attention to those dangerous permissions because they can cause harm to the mobile device. Further, optimisation features can also be used to obtain the best features of the permissions.

### Machine learning process

Machine learning, known as artificial intelligence, is used to solve complex problems and reduce decision-making times for human beings. They are used in several fields, such as medicine, space exploration, engineering, lab work, aviation and more [50]. In this study, the machine learning approach was developed to heighten the effectiveness of malware detection. It automates the analytical model that characterises the malware behaviour process. Typically, the machine learning process involves three phases: File representation, feature selection, and classification [35]. Machine learning takes two routes: Supervised and unsupervised techniques [37]. Supervised learning uses label datasets that are classified as either malware or benign. The training dataset is evaluated by using application features with a class label. In contrast, unsupervised learning uses unlabeled datasets which are assigned to a different group known as a cluster. The large datasets are then grouped into smaller datasets with similarities. This study uses supervised and randomised datasets to evaluate permission features on detecting Android malware.

Feature optimisation is used to optimise permission features. The implementation of the optimisation approach can reduce training and testing time, reduce overfitting, and simplify malware detection. This approach is ideal for data processing and increasing the accuracy of the experiments. It is an effective malware detection system. The process begins with cleaning the dataset, which removes any irrelevant and redundant features. The next stage is to acquire data randomisation. For this, the Waikato Environment for Knowledge Analysis (WEKA) was used to evaluate the Android application. Weka is one of the machine learning software used to solve complex issues. It provides a graphical interface to display the results and different algorithms to predict and model the data [51]. applied WEKA to evaluate and improve the quality of Android software [52]. used WEKA to process the failure log data set for industrial machine components and prioritise components that are more likely to fail. In addition, WEKA provides optimum results for feature optimisation as well as for dataset filtering processes [53]. The filtering process was used to predict datasets at random. This process allows any possible biases to be removed during the experiment [19]. applied WEKA for features optimisation and malware detection. In this study, to select the best features, the optimisation technique was used. Three optimisation techniques were selected to distinguish the malware detection results. They included particle swarm optimisation (PSO), information gain, and evolutionary computation. The performance between the different classifiers was measured to determine the effectiveness of the Android malware detection.

PSO is an approach that seeks to solve problems in optimising decision-making. It was created by Kennedy and Eberhart in 1995 [54]. PSO is a swarm-intelligence-based, non-deterministic optimisation approach. It was created from the desire to simulate animal behaviour (bird swarms) [55]. This approach and the location of the particles (sample of the experiment) is alternated in the research area to achieve optimal results and resolve computational restrictions. The optimisation algorithm in PSO is capable of enhancing machine learning approaches for detecting malware [19]. The PSO applied in [19] was intended to increase malware detection performance. This study [56] used the PSO to optimise the random generation of candidate detectors and parameters because PSO enhances the ANFIS performance [55] by modifying membership functions and reducing errors. The PSO concept includes each time step, changing the velocity of a particle represented by pbest (the value of fitness) and gbest (global version). Information gain then evaluates the value of an attribute by assessing the information gain concerning the class. Evolutionary computation explores the attribute space via an evolutionary algorithm. To identify efficacy in the Android malware detection areas, the performances of five classifiers were compared. This study implemented a 10,000 sample dataset which included 5,000 malware and 5,000 benign samples.

## Experiment and results

This section describes the Android malware detection system and the evaluation process that used WEKA as the machine learning tool. This study evaluated the effectiveness of an Android malware detection system that applied static analysis techniques with machine learning approaches. A standard metric was used for detecting malware. A high true positive (TP) value was required to depict an accurate result. A TP occurs when the system correctly recognises malware as malicious. A false negative (FN) occurs when it incorrectly identifies malware as benign. A true negative (TN) occurs if the system recognises the benign programme as benign correctly, whereas false positive (FP) occurs when a benign programme is incorrectly identified as malicious. This study used the Androzoo dataset for benign applications and the Drebin dataset for malware applications. The total number of datasets involved in this experiment amounted to 10,000 samples.

The evaluation process started with the collection of the malware and benign samples. These were then combined into one database for training and testing the sets. The datasets were then labelled as malware or benign. The datasets were first pre-preprocessed and then filtered as unsupervised and randomised. The PSO, information gain, and evolutionary computation were then utilised for optimisation to obtain the best features. Tables 6–8 shows the twenty features selected from PSO, information gain and evolutionary computation approaches. Following this, the dataset that was evaluated by the five classifiers with 10-fold cross-validation was used to restrict the problem of overfitting. It is important to ensure that the dataset can be read and the technique can be facilitated in an unknown dataset [15]. Table 9 shows the result of the five machine learning classifiers used for detecting malware. They were Random Forest, MLP, kNN, J48, and Adaboost.

**Table 6:** 20 features selection of PSO.

PSO
android.permission.FACTORY_TEST
android.permission.GLOBAL_SEARCH
android.permission.INSTALL_PACKAGES
android.permission.MOUNT_UNMOUNT_FILESYSTEMS
android.permission.PERMISSION_NAME
android.permission.PERSISTENT_ACTIVITY
android.permission.READ_PHONE_STATE
android.permission.RECEIVE_BOOT_COMPLETED
android.permission.RECEIVE_SMS
android.permission.RECORD_VIDEO
android.permission.RESTART_PACKAGES
android.permission.SEND_SMS
android.permission.USE_CREDENTIALS
android.permission.WRITE_SECURE_SETTINGS
android.permission.WRITE_SMS
android.webkit.permission.PLUGIN
com.android.alarm.permission.SET_ALARM
com.android.browser.permission.READ_HISTORY_BOOKMARKS
com.android.launcher.permission.INSTALL_SHORTCUT
com.google.android.providers.gsf.permission.READ_GSERVICES

**Table 7:** 20 features selection of information gain.

Information Gain
Android.03permission.READ_PHONE_STATE
Android.permission.SEND_SMS
Android.permission.READ_SMS
Android.permission.RECEIVE_SMS
Android.permission.RECEIVE_BOOT_COMPLETED
Android.permission.WRITE_SMS
com.Android.launcher.permission.INSTALL_SHORTCUT
Android.permission.INTERNET
Android.permission.INSTALL_PACKAGES
com.Android.browser.permission.WRITE_HISTORY_BOOKMARKS
com.Android.browser.permission.READ_HISTORY_BOOKMARKS
com.Android.launcher.permission.UNINSTALL_SHORTCUT
com.lge.launcher.permission.READ_SETTINGS
com.motorola.launcher.permission.READ_SETTINGS
com.motorola.dlauncher.permission.READ_SETTINGS
com.htc.launcher.permission.READ_SETTINGS
com.motorola.launcher.permission.INSTALL_SHORTCUT
com.lge.launcher.permission.INSTALL_SHORTCUT
com.motorola.dlauncher.permission.INSTALL_SHORTCUT
com.Android.launcher.permission.READ_SETTINGS

**Table 8:** 20 features selection of evolutionary computation.

Evolutionary Computation
android.permission.ACCESS_LOCATTON_MOCK_LOCATION
android.permission.DEVICE_POWER
android.permission.FACTORY_TEST
android.permission.INSTALL_PACKAGES
android.permission.GLOBAL_SEARCH
android.permission.MOUNT_UNMOUNT_FILESYSTEMS
android.permission.PERMISSION_NAME
android.permission.PERSISTENT_ACTIVITY
android.permission.READ_PHONE_STATE
android.permission.RECEIVE_BOOT_COMPLETED
android.permission.RECEIVE_SMS
android.permission.RECORD_VIDEO
android.permission.RESTART_PACKAGES
android.permission.SEND_SMS
android.permission.USE_CREDENTIALS
android.permission.WRITE_SECURE_SETTINGS
android.permission.WRITE_SMS
android.webkit.permission.PLUGIN
com.android.alarm.permission.SET_ALARM
com.android.browser.permission.READ_HISTORY_BOOKMARKS

**Table 9:** Results of detection performance.

Optimisation Technique	Machine Learning Classifier	Performance Metric					
		Accuracy (%)	TPR (%)	Precision (%)	Recall (%)	F-Measures (%)	FPR
PSO	**Random Forest**	**91.59**	**91.6**	91.6	91.6	91.6	0.084
	MLP	91.24	91.2	91.3	91.2	91.2	0.088
	kNN	91.56	91.6	91.6	91.6	91.6	0.084
	J48	91.07	91.1	91.1	91.1	91.1	0.089
	Adaboost	89.03	89.0	89.2	89.0	89.0	0.110
Information Gain	Random Forest	90.54	90.5	90.5	90.5	90.5	0.095
	MLP	90.56	90.6	90.6	90.6	90.6	0.094
	kNN	90.6	90.6	90.6	90.6	90.6	0.094
	J48	90.35	90.4	90.4	90.4	90.3	0.097
	Adaboost	89.53	89.5	89.6	89.5	89.5	0.105
Evolutionary Computation	Random Forest	90.5	90.5	90.6	90.5	90.5	0.095
	MLP	90.49	90.5	90.5	90.5	90.5	0.095
	kNN	90.51	90.5	90.6	90.5	90.5	0.095
	J48	90.18	90.1	90.3	90.2	90.2	0.098
	Adaboost	88.55	88.6	88.7	88.6	88.5	0.115

Table 9 shows the detection performance of five Android malware detection classifiers. The performance of each classifier is measured by five performance metrics such as TPR, FPR, precision, recall, f-measure and accuracy. The table indicates that PSO performs better than information gain, evolutionary computation. Furthermore, Random Forests and kNN on PSO perform well with 91.6% TPR. For each classifier, the average TPR is above 90% except for Adabosst, which is 89%. The results showed that permission-based features and machine learning classifiers are an effective way to detect Android malware. Features optimisation also helped to identify the best possible features and enhance accuracy in Android malware detection.

### Receiver operating characteristic curve

**Fig 8:**
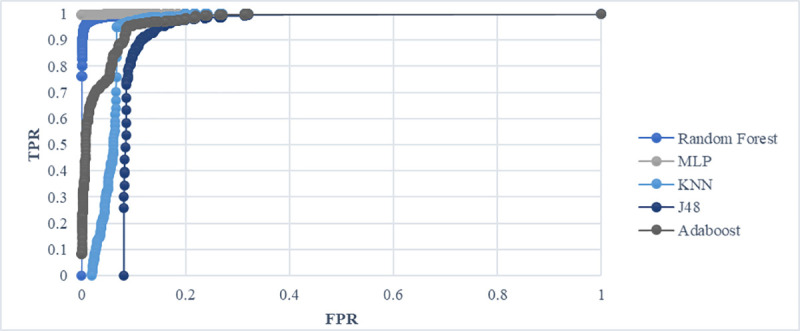
Receiver operating characteristic curve.

A receiver operating characteristic curve (ROC) is a graphical plot. It is used to represent the evaluation of performance detection in machine learning approaches. The ROC curve measures the effectiveness of the classifier prediction. The more closely the apex curve approaches the upper left corner, the more accurate the prediction. The left corner indicates that the classifier correctly detected the malware; it showed high accuracy with minimal false alarms. Fig 8 indicates that the five classifiers produced good performances based on the PSO results because the ROC curve was close to the top left corner.

### Area under a curve

The area under a curve (AUC) is used to measure the performance of the classifier with the following threshold values: 1 indicates perfect prediction, 0.9 excellent prediction, 0.8 good prediction, 0.7 mediocre prediction, and 0.6 poor prediction. Table 10 shows the evaluation of AUC, in which all the classifiers obtained excellent prediction. This demonstrates that the features chosen are capable of detecting Android malware. Random Forest attained the highest accuracy rate and was the most effective classifier for predicting malware. Random Forest is an ensemble classifier in machine learning using decision trees. A different subset of training data are constructed with an auxiliary to train each tree. This classifier is also known as the divide and conquer algorithm. It has been proven to produce highly accurate results [53] and [6] is effective in detecting malware.

**Table 10:** Evaluation of AUC.

Classifier	AUC	Level
Random Forest	0.9974	Excellent Prediction
kNN	0.9945	Excellent Prediction
J48	0.9839	Excellent Prediction
MLP	0.9672	Excellent Prediction
Adaboost	0.9605	Excellent Prediction

### Box plot analysis

**Fig 9:**
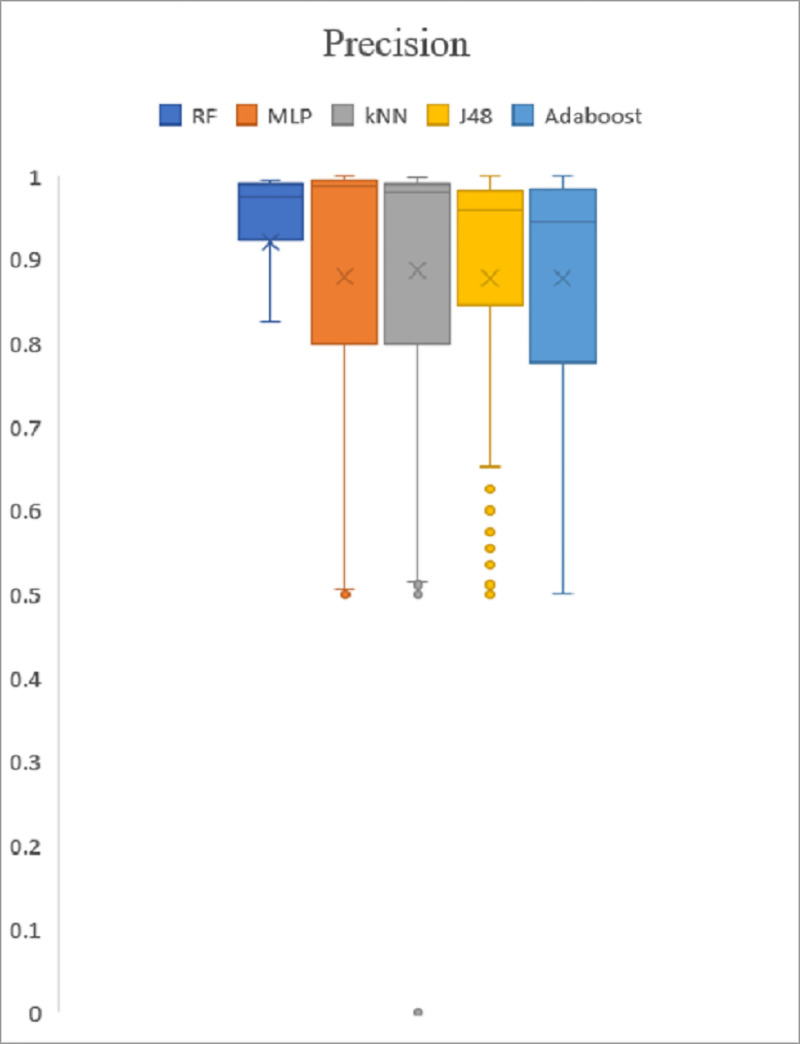
Precision.

**Fig 10:**
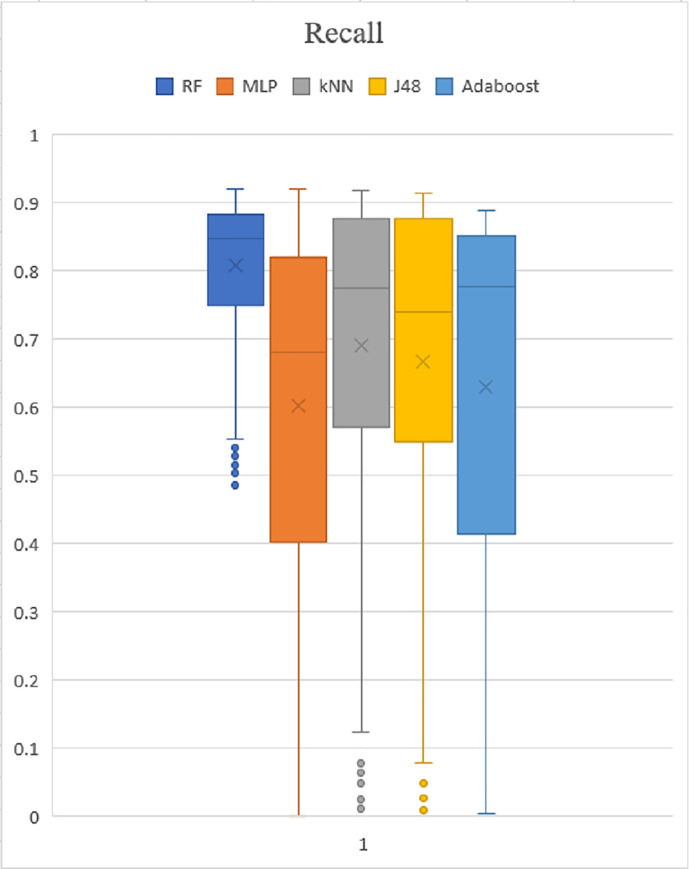
Recall.

**Fig 11:**
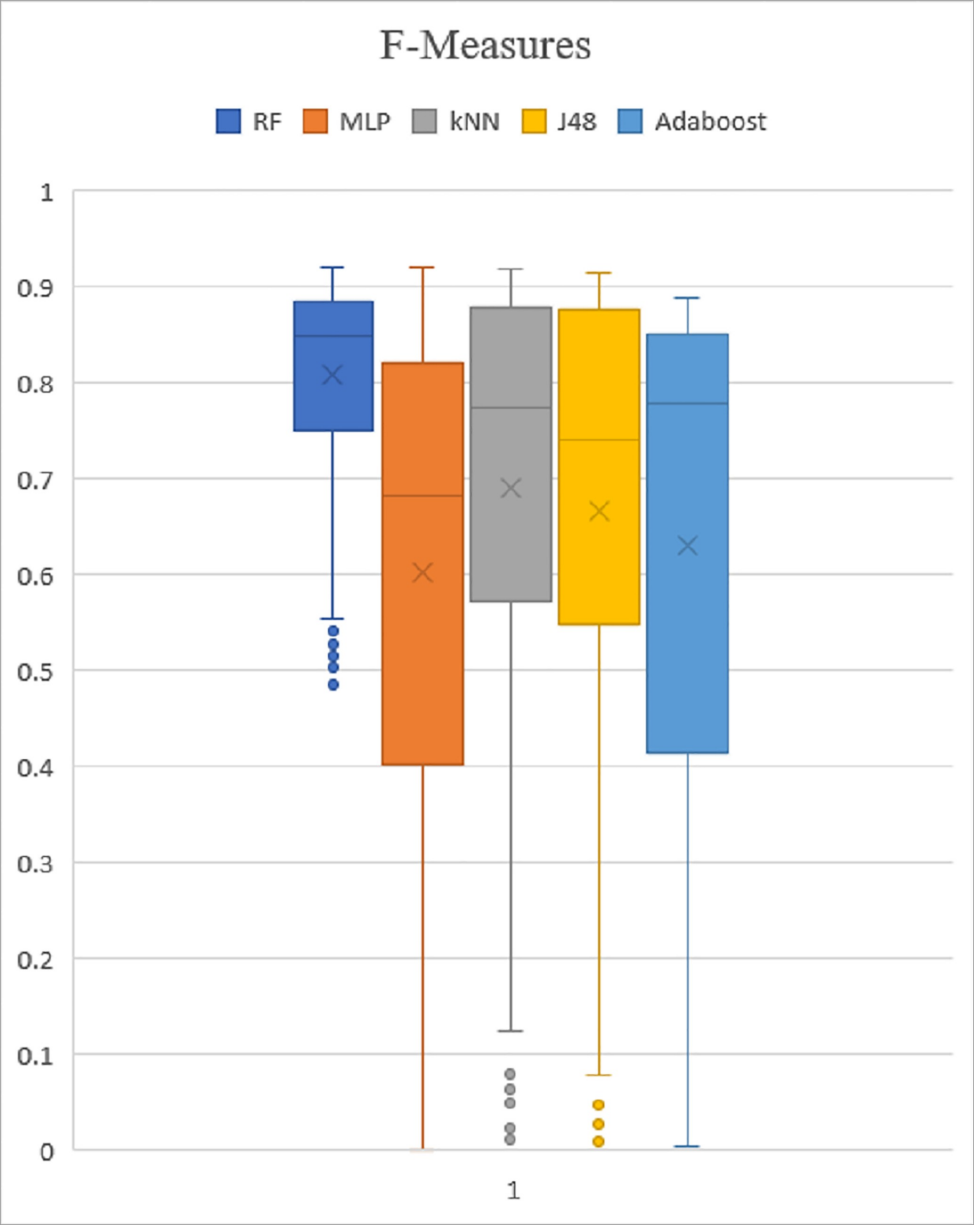
F-Measure.

The box plot analysis is applied to validate the experiment assessment based on the PSO performance metric result. All test results have been saved in a.arff file, and then converted to a.csv file. The result of precision, recall and f-measure were then produced in box plot graphs for experimental evaluation and are shown in Figs 9–11. The top whiskers demonstrate that the five classifiers achieved high precision rates of greater than 0.9%. Recall and f-measure rates also achieved high result with significantly higher than 0.9%, except for the Adaboost, the performance value is greater than 0.8%. In addition, five classifiers also show the value is going from 0.0 to 1.0. As a result, it was indicated that the performance demonstrates high precision with a high recall rate, proving that it is effective at accurately detecting malware.

## Discussion

This section discusses the results of static analysis for Android malware detection. To enhance the accuracy of Android malware detection, three optimisation techniques are used for features selection–PSO, information gain and evolutionary computation. Twenty features of the Android application are selected and has been evaluated by a machine learning approach with five classifiers. PSO with Random Forest classifier present the best result accuracy compare to others. Despite this, the others classifiers still consistently produces high-accuracy results in excess of 88%. The comparative analysis of the study’s findings with previous research served to emphasise the study’s relevance. Table 11 show the comparative study with related work.

**Table 11:** Comparative study with related work.

Reference	Objective	Features	Algorithm	Result
**This study**	To propose a mobile malware detection system based on static analysis	Permission-based	Particle Swarm Optimisation with 5 machine learning classifiers (RF, MLP, J48, kNN, Adaboost)	Accuracy = 91.6%
[57]	To identify those spare permissions requested and use this information in the security and privacy approach, which use static and code analysis	Permission-based, code analysis	Bayesnet, Naive Bayes, logistic regression, MLP, Ibk, K star, Decision Table, OneR, J48, RF, Random Tree.	Accuracy = 91.95%
[9]	Providing an efficient classification model to detect mobile malware or mobile malware risk factors	Permission and API calls	RF, J48, Random Tree, kNN and Naive Bayes.	F-measure = 94.3%
[58]	To identify the best set of permission and intent in malware detection	Permission and intent	SVM, Naïve Bayes and Random Forest	Accuracy = 94.73%

[51] has shown 91.95% accuracy in malware detection. However, only 7400 datasets were used compared with 10,000 data sets in this study [9]. applied information gain as features optimisation, and Random Forest achieved the highest F-measure result, 94.3% in malware detection. However, there is only an F-measure result is present. [58] applied information gain as features optimisation and indicates Random Forest is the highest accuracy result, 94.73% in malware detection. However, it involved a small dataset which is 3128, and only accuracy results are present. In contrast, this study examines three optimisation techniques for the selection of features, namely PSO, information gain and evolutionary computation. It also displays complete metric evaluation results and achieves high accuracy and low FPR. The FPR value is important in determining the value of malware that has been incorrectly predicted [35]. The results of the performance assessment show that this approach can detect well-known malware for Android. This study also used the real Android data collection and applied different classifiers to evaluate Android malware detection.

## Conclusion

As previously stated, the current study utilised a machine learning approach which was comprised of five classifiers to detect Android mobile malware. The most effective classifiers were selected, which consisted of Random Forest, MLP, kNN, J48, and Adaboost. Permission features have been utilised to evaluate the efficiency of classifiers based on TPR results. Drebin’s malware dataset includes 5,000 samples, while Androzoo’s benign dataset included 5,000 benign samples. They were then utilised to evaluate the static analysis technique. When applying the machine learning approach, the three stages of feature optimisation, training classifiers, and the evaluation of the machine learning classifiers were observed. The experiment results showed that Random Forest achieved the highest TPR of 91.6% for feature optimisation. This result proved that the machine learning classifier was able to detect Android malware.

As is the case in all research, the current study was also constrained by some limitations. First, it focused only on permission-based features. Future studies may want to extend the model by identifying more malware behaviours along with the extraction of other features, such as API calls and code analysis. These could serve as the basic input for the model. A risk assessment system could also be considered in future studies to optimise malware detection. Through the risk assessment, the risk of each permission request would then be prioritised and zoned. The prioritising and zoning practice would raise mobile users’ awareness of potential malware damage. It is hoped that the research community will consider conducting further investigations into the problem of Android malware. The input of the community would serve as a valuable reference for others interested in malware research.

## Supporting information

S1 Data (ZIP)

## References

[1] Razak M. F. A., Anuar N. B., Salleh R., and Firdaus A., “The rise of ‘malware’: Bibliometric analysis of malware study,” *J*. *Netw*. *Comput*. *Appl*., vol. 75, pp. 58–76, 2016, doi: 10.1016/j.jnca.2016.08.022

[2] G Data, “Cyber attacks on Android devices on the rise,” 2018. [Online]. Available: https://www.gdatasoftware.com/blog/2018/11/31255-cyber-attacks-on-android-devices-on-the-rise.

[3] GData, “Mobile Malware Report -no let-up with Android malware,” 2019. [Online]. Available: https://www.gdatasoftware.com/news/2019/07/35228-mobile-malware-report-no-let-up-with-android-malware.

[4] Mcaffee, “McAfee Mobile Threat Report Q1,” 2019. [Online]. Available: https://www.mcafee.com/enterprise/en-us/assets/reports/rp-mobile-threat-report-2019.pdf.

[5] Kakavand M., Dabbagh M., and Dehghantanha A., “Application of machine learning algorithms for android malware detection,” *ACM Int*. *Conf*. *Proceeding Ser*., pp. 32–36, 2018, doi: 10.1145/3293475.3293489

[6] Narudin F. A., Feizollah A., Anuar N. B., and Gani A., “Evaluation of machine learning classifiers for mobile malware detection,” *Soft Comput*., vol. 20, no. 1, pp. 343–357, 2016, doi: 10.1007/s00500-014-1511-6

[7] Alzaylaee M. K., Yerima S. Y., and Sezer S., “DL-Droid: Deep learning based android malware detection using real devices,” *Comput*. *Secur*., vol. 89, 2020, doi: 10.1016/j.cose.2019.101663

[8] Buchanan W. J., Chiale S., and Macfarlane R., “A methodology for the security evaluation within third-party Android Marketplaces,” *Digit*. *Investig*., vol. 23, pp. 88–98, 2017, doi: 10.1016/j.diin.2017.10.002

[9] Alazab M., Alazab M., Shalaginov A., Mesleh A., and Awajan A., “Intelligent mobile malware detection using permission requests and API calls,” *Futur*. *Gener*. *Comput*. *Syst*., vol. 107, pp. 509–521, 2020, doi: 10.1016/j.future.2020.02.002

[10] Qamar A., Karim A., and Chang V., “Mobile malware attacks: Review, taxonomy & future directions,” *Futur*. *Gener*. *Comput*. *Syst*., vol. 97, pp. 887–909, 2019, doi: 10.1016/j.future.2019.03.007

[11] Saadi C., Kandrouch I., and Chaoui H., “Proposed security by IDS-AM in Android system,” 2019 I*nt*. *Conf*. *Optim*. *Appl*. *ICOA 2019*, pp. 1–7, 2019, doi: 10.1109/ICOA.2019.8727616

[12] Rehman A., Ur Rehman S., Khan M., Alazab M., and T. R. G, “CANintelliIDS: Detecting In-Vehicle Intrusion Attacks on a Controller Area Network using CNN and Attention-based GRU,” *IEEE Trans*. *Netw*. *Sci*. *Eng*., vol. 4697, no. c, pp. 1–11, 2021, doi: 10.1109/TNSE.2021.3059881

[13] Numan M. et al., “A Systematic Review on Clone Node Detection in Static Wireless Sensor Networks,” *IEEE Access*, vol. 8, pp. 65450–65461, 2020, doi: 10.1109/ACCESS.2020.2983091

[14] Amin M., Tanveer T. A., Tehseen M., Khan M., Khan F. A., and Anwar S., “Static malware detection and attribution in android byte-code through an end-to-end deep system,” *Futur*. *Gener*. *Comput*. *Syst*., vol. 102, pp. 112–126, 2020, doi: 10.1016/j.future.2019.07.070

[15] Alam S., Alharbi S. A., and Yildirim S., “Mining nested flow of dominant APIs for detecting android malware,” *Comput*. *Networks*, vol. 167, p. 107026, 2020, doi: 10.1016/j.comnet.2019.107026

[16] Comput J. P. D., Tong F., and Yan Z., “A hybrid approach of mobile malware detection in Android,” *J*. *Parallel Distrib*. *Comput*., vol. 103, pp. 22–31, 2017, doi: 10.1016/j.jpdc.2016.10.012

[17] Li J., Sun L., Yan Q., Li Z., Srisa-An W., and Ye H., “Significant Permission Identification for Machine-Learning-Based Android Malware Detection,” *IEEE Trans*. *Ind*. *Informatics*, vol. 14, no. 7, pp. 3216–3225, 2018, doi: 10.1109/TII.2017.2789219

[18] Chouhan R. R., “A Preface on Android Malware: Taxonomy, Techniques and Tools,” *Int*. *J*. *Recent Innov*. *Trends Comput*. *Commun*., no. June, pp. 1111–1117, 2017.

[19] Razak M. F. A., Anuar N. B., Othman F., Firdaus A., Afifi F., and Salleh R., “Bio-inspired for Features Optimization and Malware Detection,” *Arab*. *J*. *Sci*. *Eng*., vol. 43, no. 12, pp. 6963–6979, 2018, doi: 10.1007/s13369-017-2951-y

[20] Yan P. and Yan Z., “A survey on dynamic mobile malware detection,” *Softw*. *Qual J*, no. May 2017, pp. 891–919, 2018, doi: 10.1007/s11219-017-9368-4

[21] Liu X., Lin Y., Li H., and Zhang J., “A novel method for malware detection on ML-based visualization technique,” *Comput*. *Secur*., vol. 89, 2020, doi: 10.1016/j.cose.2019.101682

[22] Rabbani M., Wang Y. L., Khoshkangini R., Jelodar H., Zhao R., and Hu P., “A hybrid machine learning approach for malicious behaviour detection and recognition in cloud computing,” *J*. *Netw*. *Comput*. *Appl*., vol. 151, no. May 2019, p. 102507, 2020, doi: 10.1016/j.jnca.2019.102507

[23] Etaher N., Weir G. R. S., and Alazab M., “From ZeuS to zitmo: Trends in banking malware,” *Proc*. - *14th IEEE Int. Conf. Trust. Secur. Priv. Comput. Commun. Trust. 2015*, vol. 1, pp. 1386–1391, 2015, doi: 10.1109/Trustcom.2015.535

[24] Bernardi M. L., Cimitile M., Martinelli F., and Mercaldo F., “A fuzzy-based process mining approach for dynamic malware detection,” *IEEE Int*. *Conf*. *Fuzzy Syst*., 2017, doi: 10.1109/FUZZ-IEEE.2017.8015490

[25] Yan L. K. and Yin H., “DroidScope: Seamlessly reconstructing the os and dalvik semantic views for dynamic android malware analysis,” *Proc. 21st USENIX Secur. Symp*., no. January 2012, pp. 569–584, 2012.

[26] Wang S., Chen Z., Yan Q., Yang B., Peng L., and Jia Z., “A mobile malware detection method using behavior features in network traffic,” *J*. *Netw*. *Comput*. *Appl*., vol. 133, no. January, pp. 15–25, 2019, doi: 10.1016/j.jnca.2018.12.014

[27] Statista, “Development of new Android malware worldwide from June 2016 to May 2019,” 2019. [Online]. Available: https://www.statista.com/statistics/680705/global-android-malware-volume/.

[28] Nokia, “Nokia Threat Intelligence Report– 2019,” *Netw*. *Secur*., vol. 2019, no. 12, p. 4, 2019, doi: 10.1016/s1353-4858(18)30122-3

[29] Alcatel-Lucent, “Mobile malware: A network view,” 2015. [Online]. Available: https://www.blackhat.com/docs/ldn-15/materials/london-15-McNamee-Mobile-Malware-A-Network-View-wp.pdf.

[30] Salah Y., Hamed I., Nabil S., Abdulkader A., and Mostafa M. M., “Mobile Malware Detection: A Survey,” *Int*. *J*. *Comput*. *Sci*. *Inf*. *Secur*., vol. 17, no. 1, 2019.

[31] Gyamfi N. K., “Survey of Mobile Malware Analysis, Detection Techniques and Tool,” pp. 1101–1107, 2018.

[32] Enck W., Ongtang M., and McDaniel P., “On lightweight mobile phone application certification,” *Proc*. *ACM Conf*. *Comput*. *Commun*. *Secur*., no. May, pp. 235–245, 2009, doi: 10.1145/1653662.1653691

[33] Felt A. P., Chin E., S. Hanna, D. Song, and D. Wagner, “Android permissions demystified,” *Proc. ACM Conf. Comput. Commun. Secur*., no. October, pp. 627–636, 2011, doi: 10.1145/2046707.2046779

[34] Grace M., Zhou Y., Zhang Q., Zou S., and Jiang X., “RiskRanker: Scalable and accurate zero-day android malware detection,” *MobiSys’12—Proc*. *10th Int*. *Conf*. *Mob*. *Syst*. *Appl*. *Serv*., pp. 281–293, 2012, doi: 10.1145/2307636.2307663

[35] Faruki P., Ganmoor V., Laxmi V., Gaur M. S., and Bharmal A., “AndroSimilar: Robust statistical feature signature for android malware detection,” *SIN 2013—Proc*. *6th Int*. *Conf*. *Secur*. *Inf*. *Networks*, no. September 2015, pp. 152–159, 2013, doi: 10.1145/2523514.2523539

[36] Arzt S. et al., “FLOWDROID: Precise context, flow, field, object-sensitive and lifecycle-aware taint analysis for Android apps,” *ACM SIGPLAN Not*., vol. 49, no. 6, pp. 259–269, 2014, doi: 10.1145/2594291.2594299

[37] Mehtab A. et al., “AdDroid: Rule-Based Machine Learning Framework for Android Malware Analysis,” *Mob*. *Networks Appl*., vol. 25, no. 1, pp. 180–192, 2020, doi: 10.1007/s11036-019-01248-0

[38] Gibert D., Mateu C., and Planes J., “The rise of machine learning for detection and classification of malware: Research developments, trends and challenges,” *J*. *Netw*. *Comput*. *Appl*., p. 102526, 2020, doi: 10.1016/j.jnca.2019.102526

[39] Naway A. and Li Y., “A Review on The Use of Deep Learning in Android Malware Detection,” *Int*. *J*. *Comput*. *Sci*. *Mob*. *Comput*., vol. 7, no. 12, pp. 42–58, 2018.

[40] Jerlin M. A. and Marimuthu K., “A New Malware Detection System Using Machine Learning Techniques for API Call Sequences,” *J*. *Appl*. *Secur*. *Res*., vol. 13, no. 1, pp. 45–62, 2018, doi: 10.1080/19361610.2018.1387734

[41] Sarmah Abhijit, “Intrusion Detection Systems: Definition, Need and Challenges,” 2019. [Online]. Available: https://www.sans.org/reading-room/whitepapers/detection/intrusion-detection-systems-definition-challenges-343.

[42] Kosa J. A., “Ashish Kumar Luhach First International Conference on Sustainable Technologies for Computational Intelligence,” 2019.

[43] Feizollah A., Anuar N. B., Salleh R., Suarez-Tangil G., and Furnell S., “AndroDialysis: Analysis of Android Intent Effectiveness in Malware Detection,” *Comput*. *Secur*., vol. 65, pp. 121–134, 2017, doi: 10.1016/j.cose.2016.11.007

[44] Razak M. F. A., Anuar N. B., Salleh R., Firdaus A., Faiz M., and Alamri H. S., “‘Less Give More’: Evaluate and zoning Android applications,” *Meas*. *J*. *Int*. *Meas*. *Confed*., vol. 133, pp. 396–411, 2018.

[45] Firdaus A., Anuar N. B., Razak M. F. A., and Sangaiah A. K., “Bio-inspired computational paradigm for feature investigation and malware detection: interactive analytics,” *Multimed*. *Tools Appl*., vol. 77, no. 14, pp. 17519–17555, 2018, doi: 10.1007/s11042-017-4586-0

[46] Peiravian N. and Zhu X., “Machine learning for Android malware detection using permission and API calls,” *Proc*.*—Int*. *Conf*. *Tools with Artif*. *Intell*. *ICTAI*, pp. 300–305, 2013, doi: 10.1109/ICTAI.2013.53

[47] Google Developers, “Manifest.permission,” 2020. [Online]. Available: https://developer.android.com/reference/android/Manifest.permission#READ_PHONE_NUMBERS. [Accessed: 16-Jan-2020].

[48] Allix K., Bissyandé T. F., Klein J., and Le Traon Y., “AndroZoo: Collecting millions of Android apps for the research community,” *Proc. - 13th Work. Conf. Min. Softw. Repos. MSR* *2016*, pp. 468–471, 2016, doi: 10.1145/2901739.2903508

[49] Arp D., Spreitzenbarth M., Hübner M., Gascon H., and Rieck K., “Drebin: Effective and Explainable Detection of Android Malware in Your Pocket,” *NDSS*, no. August, 2014, doi: 10.14722/ndss.2014.23247

[50] Kumar N., Kharkwal N., Kohli R., and Choudhary S., “Ethical aspects and future of artificial intelligence,” 2016 1st Int. *Conf*. *Innov*. *Challenges Cyber Secur*. *ICICCS 2016*, no. Iciccs, pp. 111–114, 2016, doi: 10.1109/ICICCS.2016.7542339

[51] Chandra K., Kapoor G., Kohli R., and Gupta A., “Improving software quality using machine learning,” *2016 1st Int. Conf*. *Innov. Challenges Cyber Secur. ICICCS 2016*, no. Iciccs, pp. 115–118, 2016, doi: 10.1109/ICICCS.2016.7542340

[52] Lima E., Gorski E., Loures E. F. R., Portela Santos E. A., and Deschamps F., “Applying machine learning to AHP multicriteria decision making method to assets prioritization in the context of industrial maintenance 4.0,” *IFAC-PapersOnLine*, vol. 52, no. 13, pp. 2152–2157, 2019, doi: 10.1016/j.ifacol.2019.11.524

[53] Firdaus A., Anuar N. B., Karim A., and Razak M. F. A., “Discovering optimal features using static analysis and a genetic search based method for Android malware detection,” *Front*. *Inf*. *Technol*. *Electron*. *Eng*., vol. 19, no. 6, pp. 712–736, 2018, doi: 10.1631/FITEE.1601491

[54] Eberhart R. and Kennedy J., “New optimizer using particle swarm theory,” *Proc*. *Int*. *Symp*. *Micro Mach*. *Hum*. *Sci*., pp. 39–43, 1995, doi: 10.1109/mhs.1995.494215

[55] Afifi F., Anuar N. B., Shamshirband S., and Choo K. K. R., “DyHAP: Dynamic Hybrid ANFIS-PSO approach for predicting mobile malware,” *PLoS One*, vol. 11, no. 9, pp. 1–21, 2016, doi: 10.1371/journal.pone.0162627 27611312 PMC5017788

[56] Adebayo O. S. and Aziz N. A., “Improved Malware Detection Model with Apriori Association Rule and Particle Swarm Optimization,” *Secur*. *Commun*. *Networks*, vol. 2019, 2019, doi: 10.1155/2019/2850932

[57] Arslan R. S., Dogru I. A., and Barisci N., “Permission-Based Malware Detection System for Android Using Machine Learning Techniques,” *Int*. *J*. *Softw*. *Eng*. *Knowl*. *Eng*., vol. 29, no. 1, pp. 43–61, 2019, doi: 10.1142/S0218194019500037

[58] Khariwal K., Singh J., and Arora A., “IPDroid: Android malware detection using intents and permissions,” *Proc*. *World Conf*. *Smart Trends Syst*. *Secur*. *Sustain*. WS4 2020, pp. 197–202, 2020, doi: 10.1109/WorldS450073.2020.9210414

